# Análisis del sellado radicular utilizando azul de metileno en distintas técnicas de obturación endodóntica. Revisión de la literatura

**DOI:** 10.21142/2523-2754-1002-2022-110

**Published:** 2022-06-27

**Authors:** Betty Machaca Albino, Ebingen Villavicencio Caparó, Luis Armando Pacheco Ramírez, Carla Miranda Miranda

**Affiliations:** 1 División de Odontopediatría, Universidad Mayor de San Andrés. La Paz, Bolivia. bettychuletitas@hotmail.com, cmiranda@umsa.bo Universidad Mayor de San Andrés División de Odontopediatría Universidad Mayor de San Andrés La Paz Bolivia bettychuletitas@hotmail.com cmiranda@umsa.bo; 2 Carrera de Odontología, Universidad Católica de Cuenca. Cuenca, Ecuador. evillavicencioc@ucacue.edu.ec Universidad Católica de Cuenca Carrera de Odontología Universidad Católica de Cuenca Cuenca Ecuador evillavicencioc@ucacue.edu.ec; 3 División de Endodoncia, Universidad Mayor de San Andrés. La Paz, Bolivia. Endo_lap@hotmail.com Universidad Mayor de San Andrés División de Endodoncia Universidad Mayor de San Andrés La Paz Bolivia Endo_lap@hotmail.com

**Keywords:** azul de metileno, microfiltraciones de colorantes, obturación del conducto radicular, microfiltración endodóntica, methylene blue, dye microleakage, root canal filling, endodontic microleakage

## Abstract

Uno de los mayores inconvenientes en la obturación endodóntica es el problema del sellado radicular insuficiente, debido a la microfiltración de bacterias hacia el conducto. El objetivo de la investigación fue realizar una revisión narrativa de las características del empleo del azul de metileno como evaluador de microfiltraciones en estudios que compararon la calidad del sellado radicular con distintas técnicas de obturación endodóntica. El método de investigación empleado fue de revisión de la literatura. El azul de metileno fue utilizado en estudios experimentales *in vitro*, los cuales compararon técnicas de obturación como la condensación lateral, gutapercha inyectable en frío, termomecánica, entre otras. Las piezas dentarias selladas se sumergieron en un tinte de azul de metileno de 1% al 5% de concentración. Este procedimiento permitió reevaluar las muestras en distintos periodos de tiempo. En conclusión, para realizar pruebas de penetración de empastes endodónticos, el azul de metileno es una opción fácil de emplear además de confiable.

## Abstract (en)

One of the biggest drawbacks in endodontic filling is the problem of insufficient root sealing, due to the microleakage of bacteria into the canal. The objective of the research was to carry out a narrative review of the characteristics of the use of methylene blue as an evaluator of microleakage in studies that compared the quality of root sealing with different endodontic filling techniques. The research method used was literature review. Methylene blue was used in experimental in vitro studies, which compared filling techniques such as lateral condensation, cold injectable gutta-percha, thermomechanics, among others, the sealed dental pieces were immersed in a 1% methylene blue dye at 5% concentration. This procedure allowed reevaluating the samples in different periods of time. In conclusion, for endodontic filling penetration testing, methylene blue is an easy-to-use and reliable option.

## INTRODUCCIÓN

Actualmente, el número de tratamientos endodónticos ha aumentado debido a que la población opta cada vez más por la preservación de sus piezas dentarias. El tratamiento endodóntico tiene la finalidad de la preservación de la pieza dentaria mediante la obturación del conducto radicular, debido a que logra un adecuado sellado hermético que evita la reinfección y la presencia de microorganismos en su interior ^1^. La literatura ofrece una diversidad de opiniones sobre la mejor forma para lograrlo, pues se cuenta en la actualidad con muchas técnicas, dispositivos y materiales para lograr la obturación del conducto radicular. 

Los reportes indican que casi un 60% de los fracasos en el tratamiento endodóntico están asociados con deficiencias en la obturación que permiten la microfiltración ^2^. A lo largo del tiempo, se realizaron distintas investigaciones enfocadas en encontrar el mejor método para cuantificar y cualificar la microfiltración apical; para ello, se tomaron en cuenta variables como las técnicas de obturación aplicadas y los cementos selladores empleados ^2^. La técnica o sistema que se emplea dependerá de los conocimientos, la destreza, los recursos y los materiales, así como también de la compleja y variada anatomía de conductos radiculares en la que se esté trabajando ^3^.

El uso de tintes orgánicos es uno de los métodos más antiguos de evaluación de microfiltración, el cual representa una técnica sencilla, económica y sigue siendo hoy en día una de las más populares. Por esa razón, el uso de tinciones para determinar la calidad del sellado apical *in vitro* es un método frecuentemente utilizado por su facilidad y sensibilidad. La penetración de colorantes nos indica el espacio que queda entre la pared del conducto radicular y el material obturador ^4^.

Existen diferentes tipos de tintes, entre ellos azul de metileno, tinta china negra, azul brillante, verde brillante, fucsia básica, hematoxilina, eosina y rodamina B. Los más empleados regularmente son los dos primeros ^5^. Otros fluidos empleados, pero menos frecuentemente, son la glucosa y el nitrato de plata. 

En el ámbito científico al azul de metileno se le conoce como cloruro de metiltionina, tiene pH de 4,7; su tamaño molecular es pequeño (0,37 micras); su molécula es muy volátil y se evapora a las 72 horas, tiene una tensión superficial muy baja y el colorante tiene cierta capacidad desmineralizante.

El presente estudio tiene por finalidad aportar información actualizada sobre el empleo del azul de metileno como método para evaluar microfiltraciones. A pesar de las observaciones y críticas sobre el modelo de penetración por tinte, el azul de metileno sigue siendo el elemento más utilizado para las pruebas de penetración por colorantes en las últimas décadas; por tanto, es una herramienta aún vigente y de uso común para medir la calidad del sellado radicular ^6^. 

La importancia de las metodologías en estudios de microfiltraciones endodónticas permite tener una mejor comprensión del éxito de la terapia endodóntica con base en la comparación de los procedimientos empleados en diferentes investigaciones de filtración apical. Los resultados de estudios que evalúan la microfiltración con azul de metileno repercutirán en la decisión de elegir la opción más favorable para el tratamiento, y se convierten en un predictor de las posibilidades de éxito o fracaso en la realización de una endodoncia. Por último, este estudio tiene como objetivo mostrar los beneficios en relación al conocimiento de las propiedades de esta técnica, la descripción de su uso y las recomendaciones para emplearla de manera eficaz en la investigación de microfiltraciones.

## MATERIALES Y MÉTODOS

El presente estudio es una revisión narrativa que incluyó artículos científicos, de los cuales se sintetizó la información relevante mediante una recopilación crítica de información. 

Tras una evaluación exhaustiva, se seleccionó 14 referencias bibliográficas. La revisión de información se basó en la búsqueda de información en revistas indexadas en SciELO, PubMed y Google Académico. Las palabras clave incluyeron “azul de metileno”, “microfiltraciones de colorantes”, “obturación del conducto radicular” y “microfiltración endodóntica”.

En general, las fuentes consultadas provienen de estudios realizados en diferentes regiones (EE. UU., Europa, Asia y Latinoamérica). Un gran porcentaje de estudios revisados fueron investigaciones cuantitativas de tipo experimental, estudios in vitro aleatorizados, longitudinales y prospectivos.

## RESULTADOS Y DISCUSIÓN

### Métodos de evaluación de microfiltraciones

Existe una gran diversidad de los métodos empleados para evaluar el sellado de conductos. Para citar los más importantes, se cuenta con la observación de penetración de un colorante a lo largo del conducto mediante sección de las raíces, y por diafanización o transparentación de las mismas. También están la observación al microscopio electrónico de barrido de la penetración de diversas bacterias, la determinación por espectrometría de la penetración de radioisótopos o mediante una técnica de detección externa, y la valoración de la penetración de iones y del volumen de gas capaz de desplazarse por el conducto, mediante cromatografía ^6^.

### Métodos que utilizan tintes

Esta metodología utiliza la inmersión dental con varios tipos de tintes como eosina, azul de metileno, tinta china negra, tinta azul, tinta negra, tinta de dibujo, rodamina B y fucsina, entre los más importantes. El objetivo es que, al emplear la penetración de tinta en la zona apical en un modelo de estudio *in vitro*, generalmente, se evalúa el nivel de filtración significativa ^7^ en relación con los colorantes, el tamaño molecular de las partículas, el pH y la reactividad química. 

La prueba de penetración del tinte es fácil de realizar. La alta capacidad de penetración y coloración del azul de metileno en los empastes endodónticos lo convierte en el tinte más utilizado. Algunos autores prefieren combinar esta técnica con el método de filtración de fluidos, mientras que otros creen que los resultados de las pruebas bacterianas ofrecen una mejor aproximación a la realidad clínica. 

El método de penetración del tinte permite comparar la fuga apical en dos grupos bajo idénticas condiciones experimentales rigurosas y proporciona una imitación realista de las condiciones clínicas.

La evaluación de la fuga de tinte es la técnica más comúnmente utilizada, probablemente debido a su simplicidad para evaluar la fuga. Este es un método pasivo y depende del movimiento del líquido capilar. A pesar del uso generalizado de este método para evaluar las fugas, puede tener algunas limitaciones. Si no se controla con precisión, la probabilidad de encontrar errores puede aumentar. Algunas variables podrían cambiar significativamente los resultados y no se podrían obtener mediciones cuantitativas válidas ^8^.

### Características del empleo del azul de metileno

Un gran número de estudios han empleado el azul de metileno como tinte, principalmente porque es económico, fácil de manejar, además de que tiene un alto grado de tinción y un peso molecular inferior al de las toxinas bacterianas. Algunos investigadores han sugerido que el azul de metileno exhibe una fuga similar al ácido butírico, un producto metabólico microbiano con mayor penetración que la tinta china ^6^. 

La descripción de la forma en que se emplea el azul de metileno para la evaluación de microfiltraciones es muy similar entre todos los estudios consultados. El proceso empleado, por lo general, es sumergir todos los dientes en una solución acuosa de azul de metileno a temperatura ambiente, para posteriormente extraer muestras, en las cuales se examina la penetración del tinte. Las piezas dentarias, una vez selladas, son colocadas en una solución de azul de metileno al 1% ^9^ y 2 % ^10^^,^^11^, frecuentemente, pero también se evidenció que puede emplearse al 3% e incluso al 5% ^7^, aunque esto es infrecuente. No se encontraron estudios que usaran el azul de metileno en un porcentaje mayor al mencionado.

El tiempo para evaluar las microfiltraciones varió de estudio a estudio, pero generalmente fue de 24 a 48 horas ^10^, o a las 72 horas, hasta una o dos semanas ^9^^,^^12^, e inclusive periodos más largos que comprendían de 1, 3 y 6 meses ^13^. La temperatura a la que se exponían las muestras al azul de metileno fue, por lo común, de 37 °C ^10^^,^^11^, aunque también, pero poco frecuentemente, se alcanzó una temperatura de 55 °C.

La cantidad de penetración del tinte se midió seccionando transversal o longitudinalmente en milímetros, aproximadamente a 1, 3 o 5 mm desde el ápice hasta la parte más coronal de la penetración del tinte. Se menciona el uso de centrifugadoras durante cinco minutos y el empleo de técnicas de diafanización para hacer más claro o translúcido el objeto de evaluación ^7^. 

Uno de los instrumentos más empleados en la literatura es el microscopio, en especial el análisis mediante un estereomicroscopio ^7^^,^^9^^,^^10^^,^^12^, en muchas ocasiones se combinaron cámaras fotográficas o video como instrumentos para registrar las imágenes grabadas de posibles microfiltraciones en los sellados ^11^.

Algunos de estos estudios también comparaban el empleo del azul de metileno con otras técnicas como el empleo de tinta india. Los resultados evidenciaron que el azul de metileno tiene una penetración más profunda a lo largo de los rellenos del conducto radicular que la tinta india, lo cual prueba que el primero es más eficaz para revelar microfiltraciones ^14^. Pero otro estudio mostró que las penetraciones con azul de metileno y rodamina B fueron equivalentes con selladores AH Plus, EndoREZ y Polifil, pero para Endofill, Sealer 26 y Sealapex se observó una penetración de tinte significativamente menor cuando se utilizó azul de metileno.

También se reveló que el azul de metileno penetra a lo largo de los rellenos de raíces más fácilmente en espacios secos que en espacios llenos de agua, pues penetra a lo largo de espacios llenos de aire por acción capilar, mientras que en espacios llenos de agua lo hace por difusión. Kontakiotis ^6^ investigó la influencia de la hidratación en los vacíos dentro de los materiales de obturación del conducto radicular, para lo cual empleó un modelo de transporte del fluido y penetración del tinte, que es aplicado para eliminar el agua de los espacios, y demostró que el azul de metileno penetraba más fácilmente en espacios secos que en los llenos de agua. 

El tinte tiene algunas desventajas, como la disolución durante los procesos de desmineralización y limpieza; además, es difícil observar su punto de máxima penetración en algunos casos. Por ese motivo, varios estudios han sugerido que se utilice rodamina B en lugar del azul de metileno ^6^.

En el método de extracción o disolución del tinte de azul de metileno, las ventajas que presenta su fácil empleo se hacen evidentes, siempre y cuando la ejecución cuidadosa de los protocolos establecidos sea cumplida. Otros sistemas para evaluar microfiltraciones pueden resultar ser más precisos, pero su costo elevado dificulta su utilización en la práctica de investigación endodóntica ^3^.

## CONCLUSIONES

Las conclusiones más importantes a las que se llegó en el presente estudio, tras la revisión de los diferentes métodos de empleo azul de metileno en estudios que evalúan la calidad del sellado radicular en distintas técnicas de obturación endodóntica para evaluar la extensión de la microfiltración apical después de los procedimientos de obturación, en general, es que se ha comprobado, a través de estudios experimentales *in vitro*, que es un método válido y confiable para dicha tarea, ya que la prueba de penetración del tinte es fácil de realizar y su alta capacidad de penetración en los empastes endodónticos lo convierte en la técnica *gold standard* para evaluar microfiltraciones.

Los estudios de penetración de colorantes también han sido criticados por las limitaciones de la evaluación de microfiltraciones. Dado que las partículas de colorante son más pequeñas que las bacterias, la microfiltración podría sobrestimar la penetración bacteriana. Las piezas dentarias selladas se sumergen en un tinte de azul de metileno entre el 1% y el 5% de concentración. Esta técnica no destructiva permite reevaluar las muestras en periodos de tiempo que variaban desde las 24 horas hasta semanas e incluso meses, y que se seccionan longitudinalmente para medir la profundidad de penetración lineal del tinte.

## Financiamiento:

El estudio fue autofinanciado por los autores
